# A multi-scale cardiovascular system model can account for the load-dependence of the end-systolic pressure-volume relationship

**DOI:** 10.1186/1475-925X-12-8

**Published:** 2013-01-30

**Authors:** Antoine Pironet, Thomas Desaive, Sarah Kosta, Alexandra Lucas, Sabine Paeme, Arnaud Collet, Christopher G Pretty, Philippe Kolh, Pierre C Dauby

**Affiliations:** 1 University of Liege (ULg), GIGA-Cardiovascular Sciences, Liege, Belgium

**Keywords:** End-systolic pressure-volume relationship, Time-varying elastance, Cardiovascular system

## Abstract

**Background:**

The end-systolic pressure-volume relationship is often considered as a load-independent property of the heart and, for this reason, is widely used as an index of ventricular contractility. However, many criticisms have been expressed against this index and the underlying time-varying elastance theory: first, it does not consider the phenomena underlying contraction and second, the end-systolic pressure volume relationship has been experimentally shown to be load-dependent.

**Methods:**

In place of the time-varying elastance theory, a microscopic model of sarcomere contraction is used to infer the pressure generated by the contraction of the left ventricle, considered as a spherical assembling of sarcomere units. The left ventricle model is inserted into a closed-loop model of the cardiovascular system. Finally, parameters of the modified cardiovascular system model are identified to reproduce the hemodynamics of a normal dog.

**Results:**

Experiments that have proven the limitations of the time-varying elastance theory are reproduced with our model: (1) preload reductions, (2) afterload increases, (3) the same experiments with increased ventricular contractility, (4) isovolumic contractions and (5) flow-clamps. All experiments simulated with the model generate different end-systolic pressure-volume relationships, showing that this relationship is actually load-dependent. Furthermore, we show that the results of our simulations are in good agreement with experiments.

**Conclusions:**

We implemented a multi-scale model of the cardiovascular system, in which ventricular contraction is described by a detailed sarcomere model. Using this model, we successfully reproduced a number of experiments that have shown the failing points of the time-varying elastance theory. In particular, the developed multi-scale model of the cardiovascular system can capture the load-dependence of the end-systolic pressure-volume relationship.

## Background

Since the experiments of Suga and Sagawa [1], the concept of time-varying elastance (also termed “time-varying pressure-volume ratio”) has been extensively used by clinicians and engineers to simply and accurately represent ventricular function. This powerful concept states that ventricular pressure and volume can be related at any moment of the cardiac cycle by means of an activation function. This function, once normalized with respect to time and amplitude, is able to represent any loading condition of the ventricle. At the end of cardiac ejection (systole), this pressure-volume ratio is called the “end-systolic pressure-volume relationship” (ESPVR). The slope of the ESPVR has been widely used as a load-independent index of ventricular contractility.

Thanks to its simplicity, the time-varying elastance theory has been used in many lumped mathematical models of the cardiovascular system. This theory has been extended to better reproduce the diastolic properties of the heart [2,3]. A great advantage of this concept is that it allows fast model simulations, enabling the large number of model runs needed to identify model parameters and design patient-specific models for use at the bedside [4,5].

However, many criticisms have been leveled against the time-varying elastance concept. First, its biggest advantage, namely that it allows a simple relationship between ventricular pressure and volume, is also its biggest drawback. Indeed, this *ad hoc* approach does not consider the fact that cardiac muscle contraction begins at a microscopic scale. Second, more recent experiments have shown the end-systolic pressure-volume relationship to be more parabolic than linear in shape [6-8]. Some researchers subsequently modified the time-varying elastance theory to include various nonlinear pressure-volume relationships [9,10]. Third, instantaneous ventricular pressure has also been shown to be negatively dependent on instantaneous flow out of the ventricle, an effect that has been termed the “internal resistance” of the ventricle [11-13]. These authors also added their *ad hoc* modifications to the time-varying elastance theory to account for this resistive effect by including a flow term in the pressure-volume relationship. Finally, the relationship between ventricular pressure and volume has been demonstrated to depend on the mechanical load exerted on the ventricle [14]. This result implies the load-dependence of the ESPVR, *i.e.* that the ESPVR is not unique. This effect cannot be accounted for by any modification of the time-varying elastance theory.

These objections have lead many authors to gain deeper knowledge of the fundamental mechanisms underlying cardiac contraction. For example, Burkhoff [8] described a ventricular model, with its contraction based on chemical mechanisms initiated by a time-varying intracellular calcium concentration. Negroni and Lascano [15] conceived a muscle model based on the same type of chemical pathways. This muscle model has been inserted into different ventricle models [15] and even full cardiovascular system (CVS) models [16]. In these studies, attention was more focused on the microscopic events happening during contraction while, to our knowledge, no study investigated the influence of such microscopic models on macroscopic hemodynamic variables such as volume and pressure. However, these two variables are the ones that have been used by experimental researchers to underline the limitations of the time-varying elastance theory.

The goal of the present work is thus to implement a multi-scale CVS model, taking into account the physiological origin of cardiac contraction at the cellular level, that will provide answers to the objections formulated against the time-varying elastance theory. This implementation will allow a critical comparison of this model with the time-varying elastance model.

In the following sections, we describe how we assembled the multi-scale CVS model from existing micro and macroscopic models of myocyte contraction and the circulatory system. Then, the model parameters are identified to reproduce the hemodynamics of a normal dog. Finally, experimental protocols that have shown the limitations of the time-varying elastance theory, including the load-dependence of the ESPVR, are reproduced with the model, demonstrating that these effects are correctly captured.

## Methods

**Figure 1:**
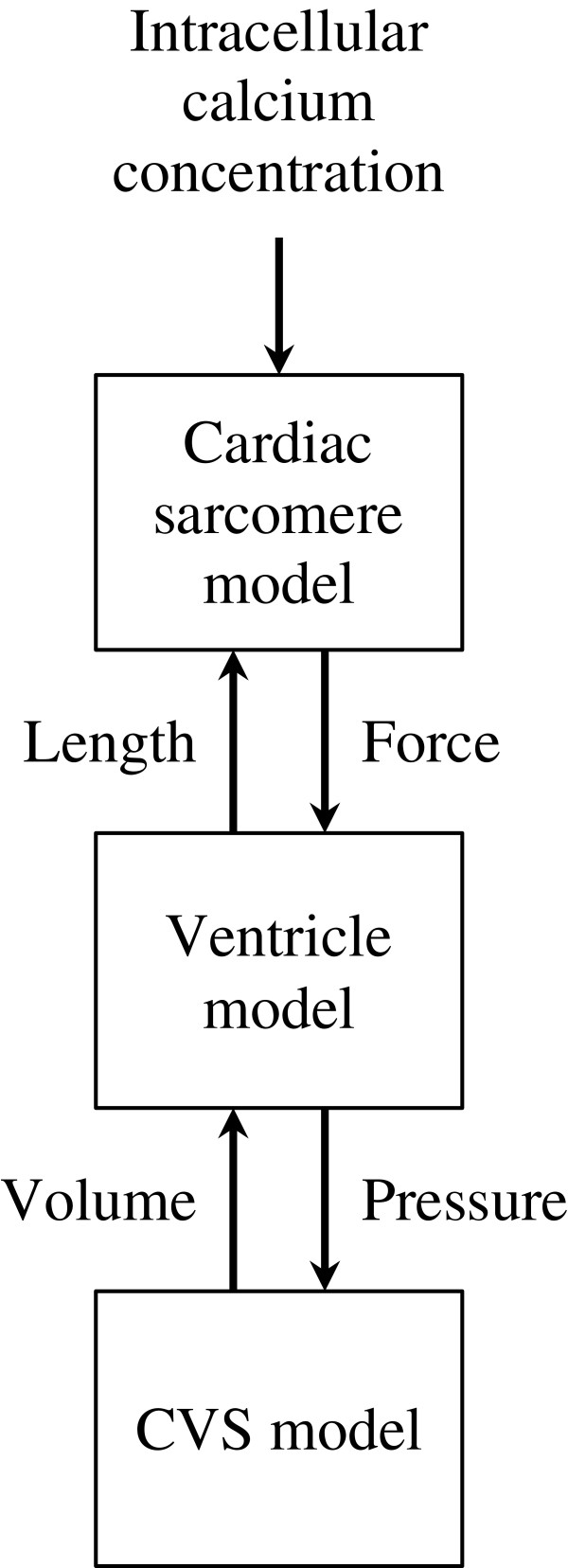
Interconnection of the three models with inputs and outputs of each model.

The multi-scale cardiovascular system model developed in this work was assembled from three existing models: (1) a cardiac sarcomere model, (2) a ventricle model and (3) a CVS model (Figure 1). This section describes how the models are interconnected, while the following subsections contain descriptions of the models.

In this work, cellular electrophysiology is not described in detail. Instead, a typical experimental curve representing how intracellular calcium concentration varies with time was used as input for a cardiac sarcomere model. This cardiac sarcomere model involves chemical equations depicting how variations in calcium concentration modify force generation. The model also describes how muscle length influences force generation. A geometrical ventricle model was built by assembling sarcomere units around a sphere. The output force of the sarcomere was then used to compute ventricular pressure, which allowed it to be inserted into a cardiovascular circulation model. Closing the loop, the circulation model dictates how much volume goes in and out of the ventricle, thereby dictating length of the individual sarcomere units. The models interconnections are presented in Figure 1.

### Calcium input

**Figure 2:**
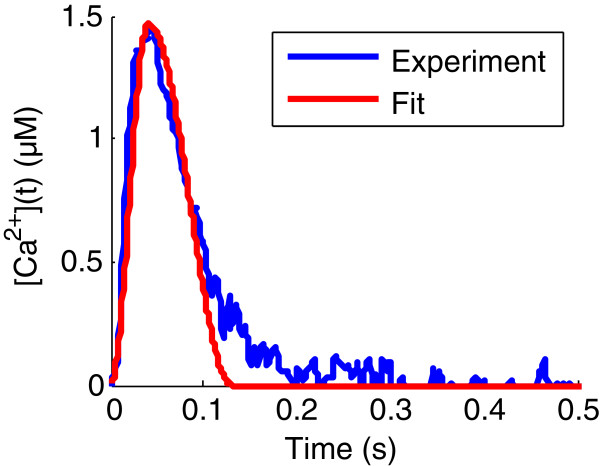
**Experimental and fit intracellular calcium concentration profiles.** Experimental intracellular calcium concentration profile (*in blue*) is taken from Yue [17]. This experimental curve is fit with two cosine branches in order to be used as input for numerical simulations (*red*).

Intracellular calcium concentration has been derived from previously published data [17] (see Figure 2). Two cosine branches were fit to the experimental profile, yielding the following expression for *Ca*^2 +^(*t*):

(1) $$\left[{\mathrm{Ca}}^{2+}\right]\left(t\right)=\{\;\begin{array}{l} \frac{{\mathrm{Ca}}_{\max}}{2}\left(1-\cos\left(\frac{\mathrm{\pi t}}{T_{1}}\right)\right)\;\mathrm{if}\;0\;\leq \;t<T_{1}\\ \frac{{\mathrm{Ca}}_{\max}}{2}\left(1+\cos\left(\frac{\pi \left(t-T_{1}\right)}{T_{2}-T_{1}}\right)\right)\;\mathrm{if}\;T_{1}\;\leq \;t<T_{2}\\ \;0\;\mathrm{otherwise} \end{array}$$

where *T*_1_ is the time at which maximum calcium concentration occurs, *T*_2_, the time at which calcium concentration goes back to zero and *Ca*_*max*_ is the maximum value of the calcium concentration. The fitting result is displayed in Figure 2 with corresponding parameter values displayed in Table 1. 

**Table 1:** Values of the model parameters

**Parameter**	**Value**	**Units**	**Source**
**Intracellular calcium concentration**			
*T*_1_	40.6	ms	Fit from [17]
*T*_2_	130.2	ms	Fit from [17]
*Ca*_*max*_	1.47	μM	Fit from [17]
**Chemical kinetics**			
*Y*_1_	39	μM s^–1^	[15]
*Z*_1_	30	s^–1^	[15]
*Y*_2_	1.3	s^–1^	[15]
*Z*_2_	1.3	s^–1^	[15]
*Y*_3_	30	s^–1^	[15]
*Z*_3_	1560	μM s^–1^	[15]
*Y*_4_	40	s^–1^	[15]
*Y*_d_	8	s μm^–2^	[15]
*T*_t_	70	μM	[15]
*B*	800	s^–1^	[15]
*h*_*c*_	0.005	μm	[15]
*L*_*a*_	1.17	μm	[15]
*R*	20	μm^–2^	[15]
**Cross-bridge parallel and elastic forces**			
*A*	1800	mN mm^–2^ μm^–1^ μM^–1^	[15]
*K*	140 000	mN mm^–2^ μm^–5^	[15]
*L*_*0*_	0.97	μm	[15]
*α*	0.5	mN mm^–2^	[15]
*β*	75	μm^–1^	[15]
**Force-length to pressure-volume conversion**			
*K*_*v*_	29.0	ml μm^–3^	Adjusted
*L*_*r*_	1.05	μm	[15]
*V*_*w*_	60.6	ml	Computed from [6]
*f*	0.217	-	Adjusted
**Hemodynamic parameters**			
*E*_*pa*_	0.953	mmHg ml^–1^	Adjusted
*E*_*pu*_	0.0302	mmHg ml^–1^	Adjusted
*E*_*ao*_	0.965	mmHg ml^–1^	Adjusted
*E*_*vc*_	0.0107	mmHg ml^–1^	Adjusted
*E*_*rv*_	2.06	mmHg ml^–1^	Computed from [6,18]
*R*_*pul*_	2.74	mmHg s ml^–1^	Computed from [6,18]
*R*_*sys*_	5.65	mmHg s ml^–1^	Computed from [6,18]
*R*_*av*_	0.152	mmHg s ml^–1^	Adjusted
*R*_*mt*_	0.0313	mmHg s ml^–1^	Adjusted
*R*_*pv*_	0.0269	mmHg s ml^–1^	Adjusted
*R*_*tc*_	0.459	mmHg s ml^–1^	Adjusted
Total blood volume	1500	ml	[19]
**Right ventricle driver function**			
*A*_1_	0.956	-	[3]
*A*_2_	0.625	-	[3]
*A*_3_	0.0180	-	[3]
*B*_1_	255	s^–2^	[3]
*B*_2_	225	s^–2^	[3]
*B*_3_	4230.0	s^–2^	[3]
*C*_1_	0.431	s	Adapted from [3]
*C*_2_	0.328	s	Adapted from [3]
*C*_3_	0.374	s	Adapted from [3]
Cardiac period	0.6	s	[3]

These values have been taken as such from the literature or have been adjusted by an iterative process (see text for details).

### Cardiac sarcomere model

The four-state model of Negroni and Lascano [15] was used to compute active force generated by sarcomeres from intracellular calcium concentration. The functioning of this model is briefly described in this section. Originally, this model did not use an input calcium concentration as described in the previous section but instead described calcium dynamics. For simplicity and rapidity of model simulation, we chose to use a calcium driver function, as done by others [8], including Negroni and Lascano [15].

**Figure 3:**
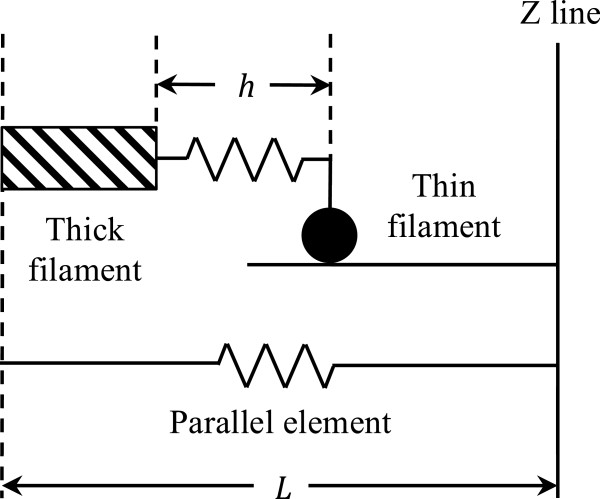
**Equivalent half-sarcomere involved in the model of Negroni and Lascano [**[15]**].** The equivalent half-sarcomere is composed of a parallel elastic element (*bottom*) and a contractile element (*top*).

The model considers only a half-sarcomere of length *L*, as shown in Figure 3. This half-sarcomere consists of an elastic element, responsible for passive force (*F*_*p*_) generation, according to the following equation:

(2) $$F_{p}=K{\left(L-L_{0}\right)}^{5}\text{,}$$

where *K* is the stiffness of the element and *L*_0_ is its resting length.

This elastic element is in parallel with a thick (myosin) filament that can bind a thin (actin) filament. The binding takes place through one “equivalent” cross-bridge (representing the average of all actual cross-bridges), whose mobile end (the black dot in Figure 3) binds actin. If the half-sarcomere shortens (*L* decreases), the equivalent cross-bridge also does (*h* decreases), and is not at equilibrium anymore. Hence, the cross-bridge will stretch, which means its length *h* will increase to reach its equilibrium value *h*_*c*_, as dictated by the following equation:

(3) $$\frac{\text{d}X}{\text{d}t}=B\left(h-h_{c}\right)=B\left(L-X-h_{c}\right)$$

where *X* = *L* − *h* and *B* is the rate at which *h* reaches its equilibrium value *h*_*c*_.

The mechanism of active force generation involves troponin (T), which, in the half-sarcomere model, goes through four different states during one calcium cycle. First, when calcium is released, it can bind troponin to form a complex, denoted TCa. This allows troponin to bind myosin, located on the thin filaments (TCa*). Afterwards, calcium can be released, while troponin is still bound to myosin (T*). Finally, troponin and myosin detach, and troponin goes back to its initial state T.

The chemical kinetics behind all these transformations are given by the following equations (see [15] for details):

(4) $$\begin{array}{l} \frac{\text{d}\left[\mathrm{TCa}\right]}{\text{d}t}{=Q}_{b}{-Q}_{a}\\ \frac{\text{d}\left[{\mathrm{TCa}}^{*}\right]}{\text{d}t}{=Q}_{a}{-Q}_{r}{-Q}_{d1}\\ \frac{\text{d}\left[T^{*}\right]}{\text{d}t}{=Q}_{r}{-Q}_{d}{-Q}_{d2} \end{array}$$

The rates involved in the previous equations are given by:

(5) $$\begin{array}{l} Q_{a}=Y_{2}\cdot {\left[\mathrm{TCa}\right]}_{\mathrm{eff}}{-Z}_{2}\cdot \left[{\mathrm{TCa}}^{*}\right]\;Q_{b}=Y_{1}\cdot \left[{\mathrm{Ca}}^{2+}\right]\cdot \left[T\right]{-Z}_{1}\cdot \left[\mathrm{TCa}\right]\\ Q_{r}=Y_{3}\cdot \left[{\mathrm{TCa}}^{*}\right]{-Z}_{3}\cdot \left[T^{*}\right]\cdot \left[{\mathrm{Ca}}^{2+}\right]\;Q_{d}=Y_{4}\cdot {\left[T^{*}\right]}^{Q_{d1}=Y_{d}\cdot {\left(\frac{dX}{dt}\right)}^{2}\cdot \left[{\mathrm{TCa}}^{*}\right]}\\ Q_{d2}=Y_{d}\cdot {\left(\frac{dX}{dt}\right)}^{2}\cdot \left[T^{*}\right] \end{array}$$

where ${\left[\mathrm{TCa}\right]}_{\mathrm{eff}}=\left[\mathrm{TCa}\right]e^{-R{\left(L-L_{a}\right)}^{2}}$ is the effective concentration of calcium bound to troponin. This effective concentration is introduced to account for the overlap between thin and thick filaments. Overlap is maximal when *L* = *L*_*a*_. *R* is a parameter controlling the curvature of the function and parameters *Y*_*i*_ and *Z*_*i*_ are reaction rates of the previous chemical reactions. Values for these parameters are displayed in Table 1. They are either taken from [15] or identified (see section “Parameter adjustment to canine data”).

Since troponin concentration does not vary with time, concentration of troponin in its initial state can be deduced from concentration of troponin in modified states ([TCa*], [*TCa*] and [T*]) by:

(6) $$\left[T\right]=T_{t}-\left[{\mathrm{TCa}}^{*}\right]-\left[\mathrm{TCa}\right]-\left[T^{*}\right]$$

where *T*_*t*_ is total troponin concentration.

In the equations describing the chemical kinetics of the sarcomeres, reaction rates can be seen to depend on *L*, the length of a half-sarcomere (through *TCa*_*eff*_), and d*L*/d*t*, the change of *L* with time (through d*X*/d*t* that influences *Q*_*d*1_ and *Q*_*d*2_). This dependence modifies the reaction rates if the sarcomere is stretched and how it is stretched, accounting for well-known force-length and force-velocity relationships [20]. Finally, active force, *F*_*b*_, is related to the concentration of troponin attached to myosin via:

(7) $$F_{b}=A\left(\left[{\mathrm{TCa}}^{*}\right]+\left[T^{*}\right]\right)\left(L-X\right)$$

where *A* is a lumped constant establishing the bridge between forces generated by a single half-sarcomere and the whole muscle unit.

Total muscle force is given by the sum of active and passive forces:

(8) $$F=F_{b}+F_{p}\text{.}$$

A series elastic element was added to model the effects of myocyte compliance. The force produced by this elastic element was defined to be:

(9) $$F_{s}=\alpha \left(e^{\beta L_{s}}-1\right)\text{.}$$

where *α* and *β* are parameters describing the shape of this passive force. The force, *F*_*s*_, and length, *L*_*s*_, of this elastic element are linked to the force, *F*, and length, *L*, of the muscle unit by:

(10) $$F=F_{s}$$

(11) $$L_{t}=L+L_{s}$$

Equation (10) has to be solved numerically to find the value of *L*.

### Ventricle model

The spherical ventricle model of Negroni and Lascano [15] was used to describe the left ventricle. This model consists of an arrangement of *N*_*c*_ half-sarcomeres on the circumference of a sphere. The number, *N*_*c*_, of half-sarcomeres can be obtained by the formula

(12) $$V_{\mathrm{mw}}=K_{v}L_{t}^{3}$$

where *V*_*mw*_ is midwall volume, the logarithmic mean of inner and outer ventricle volumes [21] and *K*_*v*_ = *N*_*c*_^3^/6*π*^2^.

The pressure inside the spherical left ventricle can be obtained by the following relationship, derived from energetic considerations [21]:

(13) $$P_{\mathrm{lv}}=5\sigma \frac{V_{w}}{V_{\mathrm{mw}}}$$

where *σ* is the average fiber stress and *V*_*w*_ is the volume of the ventricular wall. Note that the numerical factor 5 in this equation is different from the one published by Regen [21] because pressure has been converted from mN/mm^2^ to mmHg for comparison with physiological data (1 mN/mm^2^ ≈ 7.5 mmHg).

Since the force, *F*, computed in the previous section is a force per unit of muscle surface, a total force for the whole muscle unit can be computed by multiplying *F* by the reference area (*a*_*r*_) of the muscle unit:

(14) $$F_{M}=Fa_{r}\text{.}$$

Then, the average fiber stress, *σ*, can be computed by dividing this total force by the current area (*a*) of the muscle unit:

(15) $$\sigma =\frac{F_{M}}{a}=\frac{Fa_{r}}{a}\text{.}$$

Under the assumption of constant muscle unit volume, we have:

(16) $$L_{r}a_{r}=L_{t}a\text{.}$$

where *L*_*r*_ is the reference length of the muscle unit. Finally, this yields:

(17) $$\sigma =F\frac{L_{t}}{L_{r}}\text{.}$$

This, combined with Equation 13, gives the following expression for left ventricular pressure:

(18) $$P_{\mathrm{lv}}=5\frac{F}{L_{r}}\frac{V_{w}}{K_{v}L_{t}^{2}}\text{.}$$

Originally, a passive term was added to this equation to more correctly account for diastolic properties, but we chose to neglect it here after verifying that it did not greatly affect the results of the model simulations.

To derive the total length of the muscle unit from the left ventricle volume *V*_*lv*_, the fraction of wall volume enclosed in the midwall volume is assumed to be a constant [15], denoted *f*:

(19) $$f=\frac{V_{\mathrm{mw}}-V_{\mathrm{lv}}}{V_{w}}\text{.}$$

This equation is supposed to be valid for any values of the left ventricle volume, *V*_*lv*_, thus making it possible to derive the length of the muscle fiber inverting the previous equation and using Equation (12):

(20) $$L_{t}=\sqrt[{3}]{\frac{V_{w}f+V_{\mathrm{lv}}}{K_{v}}}\text{.}$$

### Cardiovascular system model

The ventricular model introduced in the previous section is then inserted into a closed-loop CVS model. Our approach is based on a model developed by Smith [2], that describes cardiac contraction by the means of the time-varying elastance theory applied to the left and right ventricles. For completeness, this initial model will be briefly described below. Then, the ventricular model presented above will replace the time-varying elastance model for the left ventricle.

**Figure 4:**
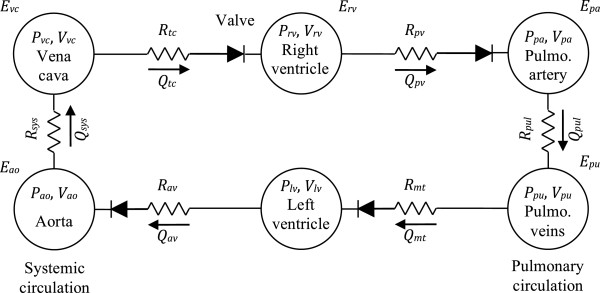
**Cardiovascular system model.** The cardiovascular system model of Smith [2] comprises six chambers, including the left and right ventricles. The left ventricle of the original model, described by a time-varying elastance, is replaced by the ventricular model described in this work.

The CVS model of Smith [2] is a lumped-parameter model consisting of six elastic chambers. These chambers represent the left (*lv*) and right (*rv*) ventricles, the aorta (*ao*), the vena cava (*vc*), the pulmonary artery (*pa*) and the pulmonary veins (*pu*). Vessels with modelled flow resistance link the six chambers. Those vessels are, respectively, the systemic (*sys*) and pulmonary (*pul*) circulations and the cardiac valves: the mitral (*mt*), aortic (*ao*), tricuspid (*tc*) and pulmonary (*pv*) valves (Figure 4). Valvular behavior is modelled with elements analogous to ideal diodes in series with additional flow resistance. 

The initial formulation of the model also included inertances, elements accounting for the inertia of the blood going through the valves [2]. We choose not to include these elements in the model, as they have been shown to have small values and to weakly affect model dynamics. Furthermore, neglecting these inertial parameters reduces uncertainty in the parameter estimation process. Finally, these parameters are difficult to measure and not well defined [22].

The model chambers are characterized by two variables: their volume (*V*) and the pressure (*P*) inside of the chamber. The two elastic chambers representing the ventricles are said to be active, which means that the relationship between the pressure and the volume is variable. More precisely, it varies between the end-diastolic pressure-volume relationship (EDPVR) and the ESPVR, respectively:

(21) $$\mathrm{EDPVR}:P_{i}\left(V_{i}\right)=P_{0,i}\left(e^{\lambda_{i}\left(V_{i}-V_{0,i}\right)}-1\right)$$

(22) $$\mathrm{ESPVR}:P_{i}\left(V_{i}\right)=E_{i}\left(V_{i}-V_{d,i}\right)$$

where *E*_*i*_ (*i* being either “*lv*” or “*rv*”) is the end-systolic elastance, *V*_*d*,*i*_, the end-systolic volume at zero pressure, *V*_0,*i*_, the end-diastolic volume at zero pressure and *P*_0,*i*_ and *λ*_*i*_ are parameters of the nonlinear relationship (21). For simplicity, we assume *V*_*d*,*i*_ and *P*_0,*i*_ to be zero, such that the EDPVR is coincident with the volume axis and the ESPVR goes through the origin.

Transition between these two extreme relationships is mediated by an activation function, varying between 0 and 1 and denoted *e*(*t*). Consequently, the pressure in a ventricle at any moment *t* of the cardiac cycle is linked to the volume by:

(23) $$P_{i}\left(t,V_{i}\left(t\right)\right)=e\left(t\right)E_{i}\left(V_{i}\left(t\right)-V_{d,i}\right)+\left(1-e\left(t\right)\right)P_{0,i}\left(e^{\lambda_{i}\left(V_{i}\left(t\right)-V_{0,i}\right)}-1\right).$$

The shape of the activation function for a dog has been described [3] as:

(24) $$e\left(t\right)=\overset{3}{\underset{j=1}{\sum }}A_{j}e^{-B_{j}{\left(t-C_{j}\right)}^{2}}\text{.}$$

Values of the constants *A*_*j*_, *B*_*j*_ and *C*_*j*_ are taken from [3] and are displayed in Table 1.

The other elastic chambers are passive, which means that their volumes and pressures are linked by a constant, the elastance, *E*.

(25) P=EV

The volume change in the six elastic chambers can be derived from the continuity equation:

(26) $$\frac{\text{d}V}{\text{d}t}=Q_{\mathrm{in}}-Q_{\mathrm{out}}$$

where *Q*_*in*_ and *Q*_*out*_ are, respectively, flow coming in and going out of the compartment. Equation (26) does not consider the heart valves, whose role is to prevent backwards flow. Hence, to correctly model the effect of the valves, a negative flow has to be replaced by a zero flow. Mathematically, it can be easily done by replacing each flow, *Q*(*t*), controlled by a valve by r(*Q*(*t*)), where r denotes the ramp function, defined as

(27) $$\text{r}\left(x\right)=\{\begin{array}{c} 0,\;\mathrm{if}\;x<0\\ x,\;\mathrm{if}\;x\;\geq \;0 \end{array}$$

Then, a positive flow is unchanged and a negative flow is replaced by 0.

A vessel element links each pair of elastic compartments of the model in Figure 4. Each of these vessels is characterized by an hydraulic resistance, denoted *R*. The relationship between flow, pressure and resistance is given by Poiseuille’s law:

(28) $$Q=\frac{P_{\mathrm{up}}-P_{\mathrm{down}}}{R}$$

where *P*_*up*_ and *P*_*down*_ represent the pressure up and downstream of the chamber, respectively. Note that the original model also takes into account the effect of the pericardium and the septum [2]. For simplicity, these effects were neglected here.

The complete cardiovascular system model of Smith *et al*. has been previously shown to be able to reproduce the major features of the cardiovascular system. It has been validated *in silico*[23], in several animal model studies [4,24,25] and is currently applied in one human study.

In our approach, and for the corresponding results discussed below, this original model is modified by considering for the left ventricle the model described in the previous section and based on the microscopic behavior of the equivalent half-sarcomere, instead of the time-varying elastance concept. This amounts to replace the description of the left ventricle by Equation (23) with Equations (1) to (11), (18) and (20). A summary of all the model equations is given in Additional file 1.

The right ventricle is still described by Equation (23), but values of *C*_*j*_ have been shifted so that left and right ventricles contract at the same time, as is the case physiologically.

### Parameter adjustment to canine data

The complete model of the CVS presented above involves a large number of parameters, which are all summarized in Table 1. The hemodynamic parameters of our model and the geometrical properties of the ventricle (*K*_*v*_, *V*_*w*_ and *f*) were numerically adjusted to match pressure and volume data from two canine experimental studies [6,18]. These are displayed in Table 2. We chose to identify these parameters because they directly influence ventricular pressure and volume (*cf*. Equations (18) and (20)) and have a macroscopic relevance. Other microscopic parameters related to the biochemical reactions and sarcomere length were kept at values obtained from literature as no data was available to adjust them. 

**Table 2:** Reference values for parameter identification

**Measurement**	**Value**	**Units**	**Source**
LV pulse pressure	119	mmHg	[6]
Mean LV pressure	66	mmHg	[6]
Aortic pressure at valve opening	115	mmHg	[6]
Aortic pressure at valve closing	121	mmHg	[6]
Mean ventricular volume	20.8	ml	[6]
Stroke volume	11.7	ml	[6]
RV pulse pressure	45.8	mmHg	[18]
Mean RV pressure	22.9	mmHg	[18]

The experimental canine pressure-volume loops published in the previous studies were taken as reference for parameter identification. More precisely, four characteristic points were manually identified on these pressure-volume loops, namely points of beginning and end of systole and diastole. This yielded a total of eight pressure-volume points that were used for parameter computation.

**Figure 5:**
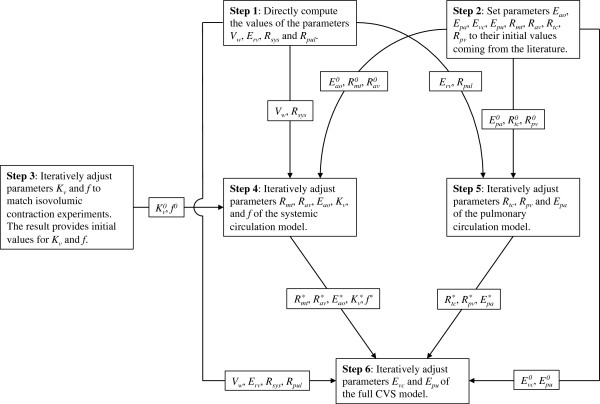
**Flow chart of the parameter estimation process.** The superscript “0” denotes initial parameter values, while the asterisk denotes converged parameter values. Parameters that are directly computed and kept constant for the rest of the identification process have no superscript.

Four parameters were directly computed from these pressure-volume points, while others were iteratively adjusted. These steps of the identification process are described in the following sections and are summarized in in Figure 5.

#### Step 1: direct parameter computation from data

Four parameters can be directly computed from the reference pressure-volume loops, the first of which is the volume of the left ventricular wall, *V*_*w*_. The end-diastolic volume makes it possible to derive the radius of the left ventricle, *R*_*lv*_, which can then be used to compute the outer ventricular volume by taking into account the ventricular wall thickness, *t*:

(29) $$V_{\mathrm{lv},\mathrm{ed}}+V_{w}=\frac{4}{3}\pi {\left(R_{\mathrm{lv}}+t\right)}^{3}\text{.}$$

where thickness was set at *t* = 0.9 cm [15]. Finally, Equation (29) can be used to compute *V*_*w*_.

Second, we simplified Equation (23) for the right ventricle by assuming *V*_*d*,*rv*_ and *P*_0,*rv*_ to be zero, which is a common assumption [5]. Equation (23) then becomes:

(30) $$P_{\mathrm{rv}}=e\left(t\right)E_{\mathrm{rv}}V_{\mathrm{rv}}\text{.}$$

Using the point of end-systole detected on the right ventricular pressure-volume loop and knowing that *e*(*t*) = 1 at end-systole (*es*) gives a direct way of computing *E*_*rv*_:

(31) $$E_{\mathrm{rv}}=\frac{V_{\mathrm{rv},\mathrm{es}}}{P_{\mathrm{rv},\mathrm{es}}}\text{.}$$

Finally, flow resistances of the systemic and pulmonary circulations, *R*_*sys*_ and *R*_*pul*_, can be directly computed using the definition of systemic vascular resistance [20] and the pulmonary equivalent, namely:

(32) $$R_{\mathrm{sys}}=\frac{{\overset{Â¯}{P}}_{\mathrm{ao}}-{\overset{Â¯}{P}}_{\mathrm{vc}}}{\mathrm{CO}}$$

(33) $$R_{\mathrm{pul}}=\frac{{\overset{Â¯}{P}}_{\mathrm{pa}}-{\overset{Â¯}{P}}_{\mathrm{pu}}}{\mathrm{CO}}$$

where the bar is used to denote the mean and *CO* represents cardiac output (the volume of blood ejected by the heart per unit time). All the right-hand side elements of Equations (32) and (33) can be estimated by inspection of the pressure-volume loops. First, cardiac output is computed by dividing stroke volume (equal to the difference between end-diastolic and end-systolic volumes) by cardiac period, which is a fixed parameter of the model. Mean arterial pressures were assumed equal to end-systolic ventricular pressures. Finally, mean vena cava pressure was assumed to be equal to the average of right ventricular pressure at the beginning and end of diastole. The same approach was applied to mean pulmonary vein pressure and left ventricular pressure.

Direct computation of the four parameters *V*_*w*_, *E*_*rv*_, *R*_*sys*_ and *R*_*pul*_ carried out as described above yielded the values displayed in Table 1.

#### Steps 2 and 3: initial values for iteratively adjusted parameters

An iterative process, described in the next section, was used to adjusted other hemodynamic parameters and geometric features of the left ventricle. Before describing this process, we will first discuss how initial values for these identified parameters were obtained.

Initial values for identified hemodynamic parameters, namely elastances of arterial and venous chambers (*E*_*ao*_, *E*_*pa*_, *E*_*vc*_ and *E*_*pu*_) and valve resistances (*R*_*mt*_, *R*_*av*_, *R*_*tc*_ and *R*_*pv*_) were taken from previously published values for pigs [5]. This initialization process is termed as “Step 2” in Figure 5.

Initial values for parameters describing geometric features of the left ventricle (*K*_*v*_ and *f*) were obtained by performing simulations of isovolumic contractions. In these experiments, the ventricle contracts while being submitted to a constant volume. An example of such simulations can be found in section "Load-dependence of the ESPVR". The interest of performing isovolumic contractions simulations is that a constant volume input suppresses the influence of the circulatory system and the related parameters, which allows the ventricular parameters to be estimated separately. Parameters *K*_*v*_ and *f* were iteratively adapted so that end-systolic and end-diastolic pressures for four different isovolumic contractions match corresponding points on the (ejecting) pressure-volume loops published by Kass [6]. The load-dependence of the ESPVR implies that maximum pressure for ejecting and isovolumic beats are different [11,12], this is why this technique was only used to get initial parameter values. The step described in this paragraph is referred to as “Step 3” in Figure 5.

#### Steps 4 to 6: numerical adjustment of other parameters

First, the CVS model was split into systemic and pulmonary circulations, by assuming constant systemic and pulmonary venous pressures [26]. (Constant venous pressures were computed as the mean values of begin- and end- diastolic ventricular pressures, see above.) The vena cava and pulmonary veins compartments become points with constant pressures, implying that the two subsystems, each composed of a ventricle and an arterial compartment, become independent. The two subsystems must share the same stroke volume, thus single stroke volume value in Table 2. Furthermore, mean ventricular volumes were assumed to be similar, hence the unique value for mean ventricular volume in Table 2.

Step 4 of the identification process was to identify parameters of the systemic subsystem (*R*_*mt*_, *R*_*av*_, *E*_*ao*_, *K*_*v*_ and *f*) with the left ventricle described by the physiological model detailed in the previous sections. Reference data are those displayed in the first 6 rows of Table 2 (mean and amplitude of left ventricle volume and pressure, plus aortic pressures at aortic valve opening and closing).

Step 5 was equivalent to step 4 applied to the pulmonary subsystem, where cardiac contraction is dictated by a time-varying elastance. Parameters *E*_*rv*_, *R*_*tc*_ and *R*_*pv*_ were adjusted so that simulated data matched reference values displayed in the 4 bottom rows of Table 2 (mean and amplitude of right ventricle volume and pressure, plus mean pulmonary artery pressure).

Finally, for step 6, the two subsystems were put back together, with venous pressures allowed to vary. The last step of the identification process was to adjust parameters *E*_*vc*_ and *E*_*pu*_ using the whole set of reference values displayed in Table 2.

In all the steps of this process, parameters were adjusted using Nelder and Mead’s simplex method [27]. All the computations were carried out using MATLAB (2011a, MathWorks, Natick, MA).

## Results

With the adjusted model parameters, we will first show (1) that the model is able to correctly reproduce the sequence of events occurring during a cardiac cycle. Then, some experimental protocols that have proven the limitations of the time-varying elastance theory will be reproduced with the model: (2) preload reduction simulations through atrial hemorrhage and increase of mitral valve resistance, (3) afterload increase simulations via increase of aortic elastance or systemic vascular resistance, (4) the same simulations with increased ventricular contractility, (5) isovolumic contraction simulations, and (6) flow-clamp simulations. These simulations allow us to investigate how well the model reproduces experimental reality. Specifically, we investigated if the model reproduced the load-dependence of the ESPVR.

### Parameter adjustment

**Figure 6:**
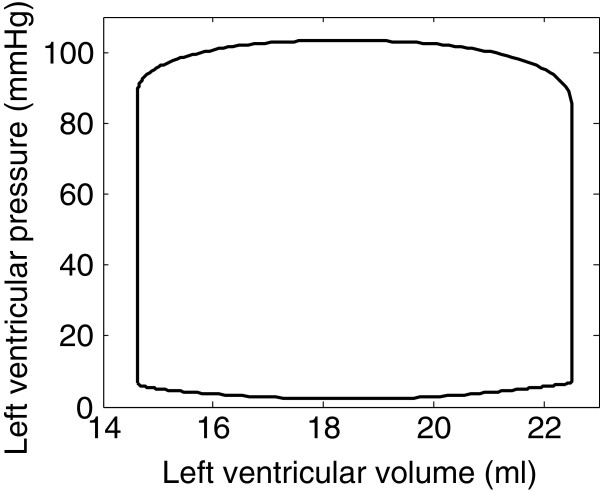
Simulated pressure-volume loop of the left ventricle.

**Figure 7:**
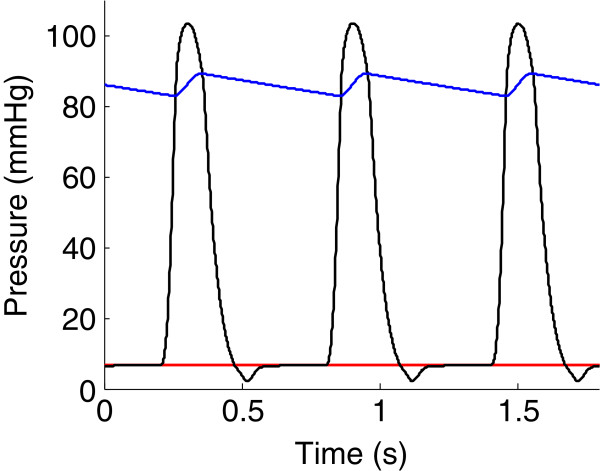
**Time course of model simulated pressures.** Simulated pressures in the pulmonary veins (*red*), left ventricle (*black*) and aorta (*blue*).

After parameter adjustment, simulated pressures and volumes were of the same order of magnitude as reference experimental results displayed in Table 2. As an example, the pressure-volume loop of the left ventricle is shown in Figure 6. Figure 7 represents simulated temporal pressure curves in the pulmonary veins, left ventricle and aorta during one heartbeat.

### Simulation of preload reduction experiments

One goal of our work was to numerically investigate the load-dependence of the end-systolic pressure-volume relationship. To do so, we numerically reproduced the experiments of Kass *et al*. [6]. These authors recorded left ventricular pressure-volume loops in six open-chest dogs with rapidly varying preload. Preload reduction was achieved by left atrial hemorrhage: a cannula was inserted in the left atrium and connected to a 1 l reservoir that was lowered to decrease left ventricle filling pressure. The preload reduction experiment consisted of lowering the reservoir for 10 to 12 s and simultaneously recording the pressure-volume loops. This technique allowed them to create a broad range of preloads, and thus to more completely characterize the ESPVR. One of their observations was that the shape of the ESPVR curve was more parabolic than linear.

**Figure 8:**
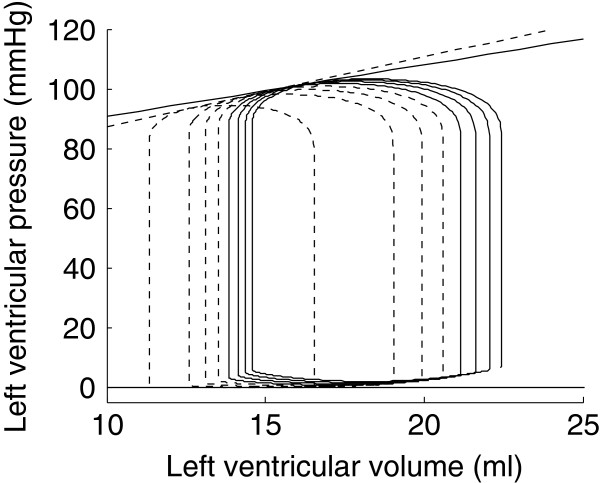
**Simulated left ventricle preload reduction experiment through atrial hemorrhage.** The first four pressure-volume loops are represented in solid lines, the last four ones, in dashed lines. Straight lines were fit to the end-systole points of the two simulated pressure-volume curves sets.

To remain as close as possible to such experimental protocols in our CVS model, an outward flow of 50 ml/s was inserted in the circulation model, at the level of the pulmonary veins. (Since the atria are not explicitly included in the model, they are merged with the pulmonary vein compartment). The model was then simulated for a further 4.8 s (i.e. 8 cycles). Resulting pressure-volume loops are shown in Figure 8.

To evaluate the linearity of the end-systolic pressure-volume relationship, we reproduced the computations carried out by Kass *et al*. [6]. Simulated pressure-volume loops were divided into two sets (one containing the first four loops, the other containing the last four) and a linear ESPVR of the form

(34) $$P_{\mathrm{es}}=E_{\mathrm{es}}\left(V_{\mathrm{es}}-V_{0}\right)$$

was computed for the two sets with an iterative process [28]. This process involved fitting a straight line to the end-systolic points, defined as points with the maximal *P*/(*V* − *V*_0_) ratio. During the first step of the process, *V*_0_ was fixed at zero. Then, coefficients of the linear regression yielded new estimates for *E*_*es*_ and *V*_0_ and the process was repeated until convergence was achieved.

**Figure 9:**
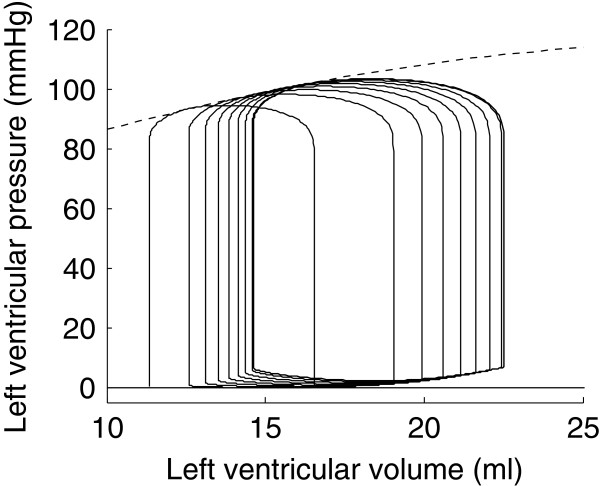
**Simulated left ventricle preload reduction experiment through atrial hemorrhage.** A parabolic curve (dashed line) was fit to the end-systole points of the simulated pressure-volume curves.

As suggested by Kass *et al*. [6], a parabolic regression of the form

(35) $$P_{\mathrm{es}}=a{\left(V_{\mathrm{es}}-V_{0}^{'}\right)}^{2}+b\left(V_{\mathrm{es}}-V_{0}^{'}\right)$$

was also computed to fit the points of end-systole. To perform this regression, the previous function was fit to points of end-systole with a nonlinear least-squares algorithm. We performed such a computation, the result of which is shown in Figure 9 and in Table 3. The curvature parameter *a* in Equation (35) was statistically different from zero with a *p* = 0.0383. 

**Table 3:** Coefficients of the linear and parabolic ESPVRs

	**Linear ESPVR**				**Parabolic ESPVR**			
	**First loops**		**Last loops**					
	*E*_*es*_	*V*_0_	*E*_*es*_	*V*_0_	*a*	p*	*b*	*V*_0_^’^
**Experiments**	6.49	−5.70	23.30	4.00	−2.68	0.005	30.00	3.90
**Simulations**	1.74	−42.2	2.33	−27.4	−0.0678	0.0383	5.62	−10.4

*Statistical significance of parameter *a*.

Experimental values come from [6].

### Load-dependence of the ESPVR

In this section, we compare the previously derived ESPVR to four other ESPVRs: one resulting from another preload reduction, two coming from afterload variations and another one resulting from isovolumic contraction simulations.

**Figure 10:**
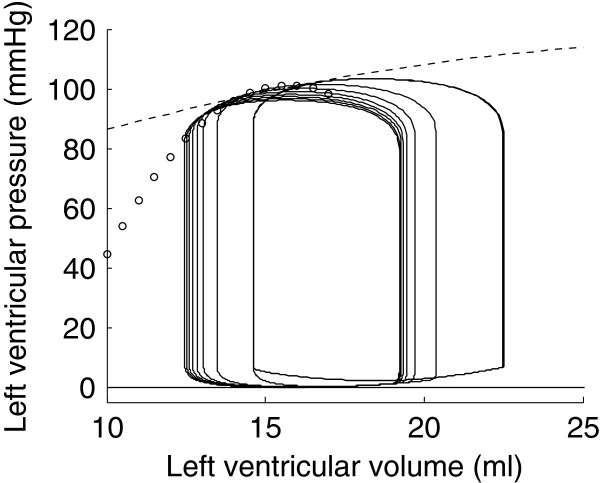
**Simulated left ventricle preload reduction experiment by increasing the mitral valve resistance.** The corresponding ESPVR is drawn with white dots. The ESPVR obtained by simulating an atrial hemorrhage is displayed in dashed line.

In the previous section, preload was varied by the means of a simulated atrial hemorrhage. Here, we simulated another preload reduction experiment by a tenfold increase of the mitral valve resistance. The result of this simulation is shown in Figure 10. Points of end-systole were computed as previously described and a parabolic ESPVR was fit to these points (white dots in Figure 10).

**Figure 11:**
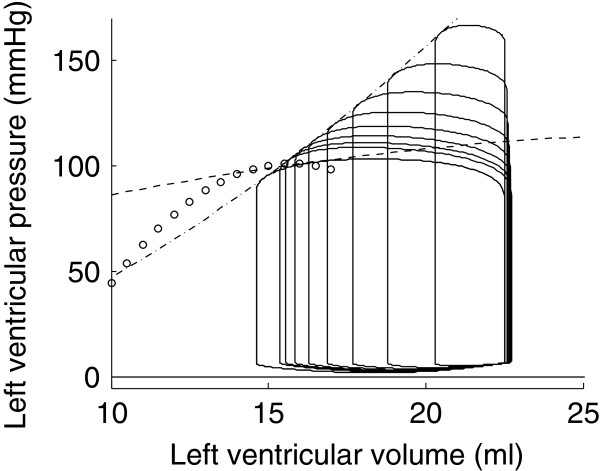
**Simulated left ventricular afterload increase by doubling aortic elastance.** The corresponding ESPVR is drawn in dash-dotted line. The ESPVRs obtained by preload reduction simulations are displayed in dashed and white-dotted lines.

Afterload can be defined as the pressure the ventricle has to overcome to eject. Doubling aortic elastance, which has the effect of increasing aortic pressure, increases afterload. The pressure-volume loops obtained by such a simulation are shown in Figure 11. Once again, we computed the ESPVR as a parabolic fit of the points of end-systole. In this case, the ESPVR was parabolic, but with a slightly positive concavity (*a* = 0.2426 mmHg/ml^2^, *p* < 0.001). This ESPVR is represented in dash-dotted line in Figure 11, along with ESPVRs obtained with preload reduction simulations.

**Figure 12:**
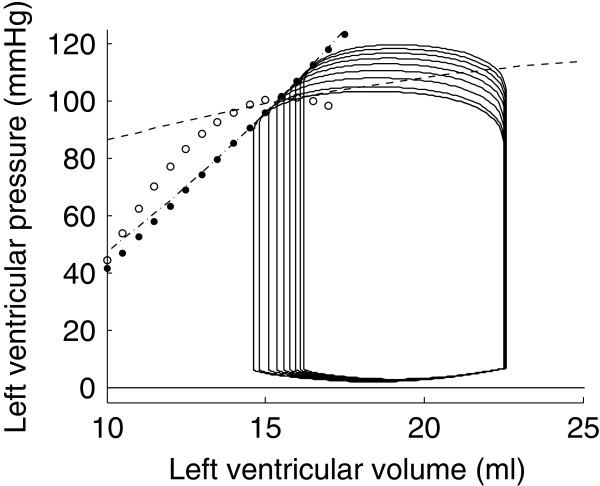
**Simulated left ventricular afterload increase by doubling systemic vascular resistance.** The corresponding ESPVR is drawn in dotted line. The ESPVRs obtained by preload reduction simulations are displayed in dashed and white-dotted lines; the ESPVR obtained by afterload increase by doubling aortic elastance is displayed in dash-dotted line.

Afterload can also be increased by doubling the value of the systemic vascular resistance in the model [23]. The simulation result is shown in Figure 12. In this figure, the ESPVR computed from points of end-systole is also displayed (black-dotted line). It was not significantly different from a straight line. (The *p*-value of the quadratic parameter *a* was *p* = 0.662.) .

To numerically reproduce the experiments of Suga and Sagawa [1] and Burkhoff [14], we also performed simulations of isovolumic contraction experiments. In an isovolumic contraction, the ventricular volume remains constant and thus only depends on the fixed volume.

**Figure 13:**
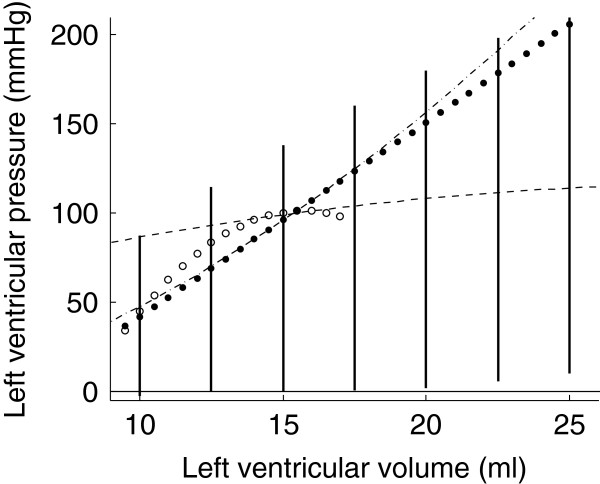
**Isovolumic contraction simulations.** Isovolumic contraction simulations are represented in bold line with four different ESPVRs generated by preload reduction simulations (dashed and white-dotted) and afterload increase simulations (dash-dotted and dotted line).

The isovolumic contraction simulations were repeated for different constant volumes, ranging from 10 ml to 25 ml. The developed pressure for each of the constant volumes is plotted in bold line in Figure 13. For comparison, the ESPVRs that were computed from the four previous load variation simulations are also shown.

### Contractility-dependence of the ESPVR

As previously explained, the ESPVR was initially proposed as a load-independent index of contractility [29]. After having examined the load-independence of the ESPVR in the previous section, we will now focus on the variations of the ESPVR due to changes in ventricular contractility.

Contractility was increased in the model by doubling the peak value of intracellular calcium concentration. In their experimental protocol, Suga *et al.*[29] increased contractility by epinephrine infusion, the effect of which is to promote calcium release by the sarcoplasmic reticulum [20]. To model this effect, we thus choose to double the value of the parameter *Ca*_*max*_ (Equation (1)), hence doubling the peak value of intracellular calcium concentration in the model.

**Figure 14:**
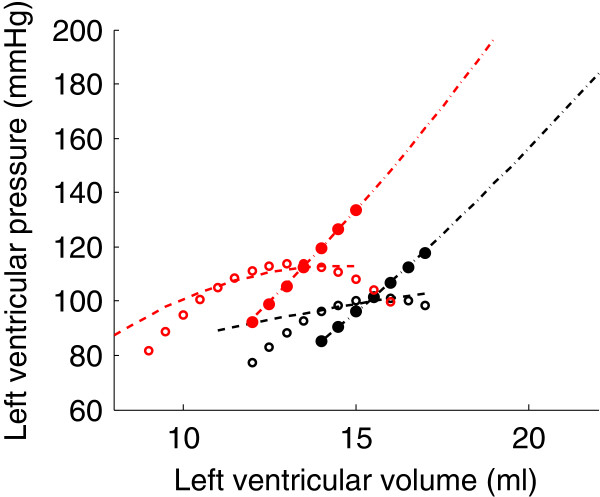
**Four pairs of different ESPVRs generated by preload reduction simulations (dashed and white-dotted) and afterload increase simulations (dash-dotted and dotted line).** Each pair of ESPVRs corresponds to basal (black) and increased (red) contractile state.

The previous preload reduction and afterload increase simulations were repeated with increased contractility. The result of these simulations is shown in Figure 14. Each one of the four ESPVRs presented in the previous section is displayed in black in this figure. The four ESPVRs obtained after increasing contractility and repeated the load variation simulations are displayed in red for comparison.

### Internal Resistance of the ventricle

Shroff [12] performed flow clamp experiments on an isolated canine heart. These experiments consisted in imposing linear ramps of ventricular volume (and consequently, constant flows out of the ventricle) and observing the effects of their timing and magnitude on ventricular pressure. The main conclusion of these authors was that, for a given ventricular volume, the bigger the flow out of the ventricle, the lower the pressure. They found that this relation between ventricular pressure and flow was linear and called the slope of the linear relation the “internal resistance” of the ventricle. This resistance was found to be approximately equal to 0.0799 mmHg s/ml (in absolute value) for a normal contractile state.

The ability of the model of Negroni and Lascano [15] to correctly reproduce various flow clamp experiments has been extensively described by these authors and hence, will not be described in detail here. In short, we also simulated flow-clamp experiments in our left ventricle model with adjusted parameters and observed, as expected, a decrease in ventricular pressure as flow out of the ventricle increased.

## Discussion

The model developed in this work accounts for the physiological, microscopic origin of cardiac contraction, making it a much more realistic model of cardiac contraction than the time-varying elastance model. But this improved realism comes with a cost: to be used, the time-varying elastance concept only requires two parameters (maximum elastance and time to peak) and one input function (the activation function) [29]. On the other hand, the model developed here requires 22 parameters (see sections “Chemical kinetics”, “Cross-bridge parallel and elastic forces” and “Force-length to pressure-volume conversion” of Table 1) and one input function (the intracellular calcium concentration). Values have to be assigned to all of these parameters, which required the development of a numerical parameter estimation scheme, as described in section “Parameter adjustment” and in Figure 5. Due to the large number of parameters, a large amount of data was required, which could not be obtained for a single animal. However, to remain as close as possible to the experiments we intended to simulate, we only used reference data coming from experiments on dogs.

After parameter adjustment, the model was able to correctly simulate the normal succession of events during a cardiac cycle. The four phases of cardiac contraction, namely filling, isovolumic contraction, ejection and isovolumic relaxation can clearly be distinguished on the pressure-volume loop of Figure 6. The timing of these phases was also physiologically correct, as shown in Figure 7. The first phase represented in Figure 7 is isovolumic contraction: after mitral valve closing, ventricular pressure increased until the aortic valve opened (crossover of ventricular and aortic pressures). Then, during ejection, the aorta filled up with blood, increasing its pressure until it exceeded the ventricular pressure. At this moment, the aortic valve closed, denoting the beginning of isovolumic relaxation. When ventricular pressure dropped below pulmonary venous pressure, the mitral valve opened, allowing filling of the ventricle. Mitral valve closure occurred when ventricular pressure rose at the initiation of a new contraction, and the cycle repeats.

Since one of the main objectives of this article was to evaluate the load-dependence of the ESPVR, this concept had to be clearly defined. We made the choice to compute ESPVRs during transient variations in ventricular volume and pressure following abrupt changes of load exerted on the ventricle. We chose this method to better agree with experimental reality. However, this method is different than some experimental procedures where ESPVR is computed only from stabilized pressures and volumes [30] or from isovolumic pressure-volume relationships [14]. Since the ESPVR is load-dependent, it is important to describe precisely how it was derived to enable correct comparisons between our model simulations and previously published experimental results.

As can be seen in Figure 8 and in Table 3, the two linear ESPVRs computed from the atrial hemorrhage experiment yielded different slopes and volume axis intercepts for each one of the two sets of loops. The same observation was made experimentally by Kass *et al*. [6], who suggested that the ESPVR could more accurately be described by a quadratic curve. The result of this computation is shown in Figure 9. As can be seen in this figure, the parabolic ESPVR obtained by atrial hemorrhage is close to linear. This may come from the insufficient variation in ventricular volume. Indeed, we had to limit our atrial hemorrhage simulation to an outward flow of 50 ml/s during 4.8 s to avoid complete emptying of the pulmonary veins. Kass *et al*. [6] used an outward flow of 80 to 100 ml/s during 10 to 12 s. Still, our parameter *a*, describing the concavity, is negative, meaning that the ESPVR is concave towards the volume axis, as experimentally determined [6,7].

The biggest assumption of the time-varying elastance theory is that the ESPVR is load-independent. It is thus assumed to be unique and to only depend on the contractile state of the heart, which makes it a powerful index of contractility. We numerically reproduced experiments that have shown the opposite, *i.e.* that the ESPVR depends on the load imposed on the ventricle. To do so, we showed that the model could exhibit many different ESPVRs. The simulation of a preload reduction experiment by atrial hemorrhage allowed us to trace a first ESPVR that was slightly parabolic and concave towards the volume axis (Figure 9). A second preload reduction experiment by increase of the mitral valve resistance yielded a clearly different ESPVR (Figure 10). Yet, the result of the two preload reduction experiments was, as expected, a shift of the pressure-volume loops towards the lower volumes. The same observation can be formulated from the results of the two afterload increase simulations (Figures 11 and 12). The ESPVRs resulting from these two simulations were similar but different from one another. They were also clearly different from the ESPVRs computed by the preload reduction simulations, reproduced in dashed and white-dotted lines in Figures 11 and 12. Also, as physiologically expected, the result of the preload increase was an increase of ventricular pressure and a decrease of stroke volume.

It has been experimentally shown that the end-systolic pressure for an ejecting beat is higher than for an isovolumic beat [14]. This is called the positive effect of ejection. This effect does not appear on our simulations since the maximal isovolumic pressures are higher than the one obtained by simulating load variations. However, as can be seen in Figure 13, one ESPVR was higher than pressures generated by isovolumic contraction simulations, at least for some values of volume. Hence, this positive effect of ejection is also present in the model. Negroni and Lascano [15] performed the same kind of experiments and were able to reproduce this experimental finding that ventricular pressure during ejection is higher than during isovolumic contraction. However, because they had not introduced their ventricle model in a model of the circulatory system, they had to create a physiological approximation of a ventricular volume curve. In our model, the ventricular volume is not imposed; it is a consequence of pressures in the other model compartments. Finally, it is also clear in Figure 13 that the pressure-volume points resulting from isovolumic contraction simulations do not fall on any of the four previously computed ESPVRs. This shows that, if one defines the ESPVR from isovolumic contraction experiments [14], the result is again different than what would be obtained by load variations.

The effect of increased contractility on the ESPVRs is a shift towards higher volumes and lower pressures (Figure 14), which is what has been experimentally observed [6,7]. Indeed, the effect of increased contractility is an increased developed pressure and a reduction in end-diastolic volume [20]. We also notice an increase in the curvature of the ESPVRs derived from preload reduction simulations. It can be seen in Figure 14 that the dashed and white-dotted ESPVRs become more curved after increasing the contractility. The same observation has been made experimentally in dogs [6]. Interestingly, we find a similar change in curvature of ESPVRs derived by afterload increases, *i.e.* the ESPVRs become more curved when contractility is increased. We found no experimental study assessing contractility-dependence of ESPVRs derived from afterload variations to compare this finding with.

Shifting of the ESPVR can be used to track variations of contractility, irrespective of how the ESPVR was derived. However, since the ESPVR is not unique, it cannot be assumed to represent an absolute measure of cardiac contractility.

As a concluding remark, please note that figures extracted from simulations and those published from experiments are not supposed to match exactly, since model parameters were adjusted to match two different experimental studies (see section “Parameter adjustment”). Furthermore, the CVS model used here is a very simple one in which the lumped properties cannot account for the rich features of experimentally measured waveforms. Additionally, only the contraction of the left ventricle was described from a microscopic point of view. This work is a first step into the microscopic description of cardiac contraction and its repercussion on hemodynamics. Hence, the goal was only for the model to be able to reproduce experimental trends, which it has achieved successfully.

## Conclusions

The model presented in this work is able to overcome the drawbacks of the time-varying elastance theory, namely its lack of physiological foundations and the load-dependence of the ESPVR. A large number of experiments that have proven the flaws of the time-varying elastance theory have been reproduced *in silico*, namely preload and afterload variation experiments, isovolumic contraction experiments, flow-clamp experiments and investigation of the effect of contractility on the ESPVR. Conclusions derived from model simulations were the same as these coming from canine experiments, *i.e.* the ESPVR is load-dependent. Consequently, when describing an ESPVR, it is essential to explain how it was derived to allow for comparison between ESPVRs.

Because the ESPVR depends on the load exerted by the vasculature on the ventricle, it cannot be considered as an intrinsic ventricular property. Additionally, the ESPVR cannot represent an absolute measure of cardiac contractility, because the shape of the ESPVR depends on how it was derived. But, instead of focusing on the absolute value of the contractility by the means of the slope of the ESPVR, it would be more reliable to speak in terms of variations of contractility from a reference state.

The work that has been done here for the left ventricle could easily be adapted to the right ventricle and, with some further modeling work, to the atria. Such a representation of the atria could be useful, since, to our knowledge, there exists no accurate time-varying elastance model applicable to the atria. Another possible improvement would be to introduce the effects of the septum and the pericardium in the circulation model. These effects have been neglected here for simplicity, but the model could easily be adapted to take them into account. With these proposed ameliorations, this microscopic contraction model would make it possible to fully describe a complete heart, composed of its four interacting chambers.

In conclusion, the multi-scale cardiovascular model developed in this work overcomes the lack of physiological grounds of the time-varying elastance theory. In addition, model simulations successfully replicate trends observed in experiments that showed the limitations the time-varying elastance theory.

## Competing interests

The authors declare that they have no competing interests.

## Authors' contributions

AP performed the model simulations and drafted the manuscript, AL, AC and SP carried out the literature review, studied the Burkhoff (1994) model and helped writing the manuscript, SK also carried out the literature review and studied the Negroni and Lascano (1996, 1999 and 2008) models, TD and PD conceived the study and participated in its design and coordination and helped to draft the manuscript, PK provided useful support about cardiovascular physiology and CP helped writing the manuscript. All authors read and approved the final manuscript.

## Supplementary Material

Additional file 1 Model Equations. Click here for file
