# Supplementary material for: A multi-scale cardiovascular system model can account for the load-dependence of the end-systolic pressure-volume relationship
Source: Biomed Eng Online. 2013 Jan 30;12:8. doi: 10.1186/1475-925X-12-8 (PMC3610305; doi:10.1186/1475-925X-12-8)
Supplement: Additional file 1 — Model Equations. [file 1475-925X-12-8-S1.pdf]

Appendix: Model Equations

Calcium Input

The model input is a time-varying intracellular calcium concentration, described by the following equation.

$$[\mathrm{Ca}^{2+}](t) = \begin{cases} \frac{\mathrm{Ca}_{max}}{2}\left(1-\cos\left(\frac{\pi t}{T_1}\right)\right) & \text{if } 0 \le t < T_1 \\ \frac{\mathrm{Ca}_{max}}{2}\left(1+\cos\left(\frac{\pi (t-T_1)}{T_2-T_1}\right)\right) & \text{if } T_1 \le t < T_2 \\ 0 & \text{otherwise} \end{cases} \quad \text{(A.1)}$$

Cardiac Muscle Model

Passive force generated by stretching the muscle unit is given by:

$$F_p = K(L - L_0)^5, \quad \text{(A.2)}$$

while active muscle force depends on two main factors: elongation of cross-bridges and concentration of attached cross-bridges. Elongation of cross-bridges is equal to $L - X$, where the return to equilibrium is governed by:

$$\frac{\mathrm{d}X}{\mathrm{d}t} = B(L - X - h_c). \quad \text{(A.3)}$$

Concentration of attached cross-bridges is linked to a chemical reaction network involving four species whose concentrations can be computed by:

$$\frac{\mathrm{d}[\mathrm{TCa}]}{\mathrm{d}t} = Q_b - Q_a$$

$$\frac{\mathrm{d}[\mathrm{TCa}^*]}{\mathrm{d}t} = Q_a - Q_r - Q_{d1} \quad \text{(A.4)}$$

$$\frac{\mathrm{d}[\mathrm{T}^*]}{\mathrm{d}t} = Q_r - Q_d - Q_{d2}$$

$$Q_a = Y_2 \cdot [\mathrm{TCa}]_{eff} - Z_2 \cdot [\mathrm{TCa}^*]$$

$$Q_b = Y_1 \cdot [\mathrm{Ca}^{2+}] \cdot [\mathrm{T}] - Z_1 \cdot [\mathrm{TCa}] \quad \text{(A.5)}$$

$$Q_r = Y_3 \cdot [\mathrm{TCa}^*] - Z_3 \cdot [\mathrm{T}^*] \cdot [\mathrm{Ca}^{2+}]$$

$$Q_d = Y_4 \cdot [\mathrm{T^*}]$$ $$Q_{d1} = Y_d \cdot \left(\frac{\mathrm{d}X}{\mathrm{d}t}\right)^2 \cdot [\mathrm{TCa^*}]$$ $$Q_{d2} = Y_d \cdot \left(\frac{\mathrm{d}X}{\mathrm{d}t}\right)^2 \cdot [\mathrm{T^*}]$$	
$$[\mathrm{TCa}]_{eff} = [\mathrm{TCa}]e^{-R(L-L_a)^2}$$ $$[\mathrm{T}] = T_t - [\mathrm{TCa^*}] - [\mathrm{TCa}] - [\mathrm{T^*}].$$	(A.6)

Finally, force generated by cross-bridges is related to concentration of attached cross-bridges and elongation of cross-bridges by:

$$F_b = A([\mathrm{TCa^*}] + [\mathrm{T^*}])(L - X).$$	(A.7)

Total force generated by the muscle unit is thus:

$$F = F_b + F_p.$$	(A.8)

A series elastic element is added to the muscle unit, whose force F_s and length L_s are given by:

$$F_s = \alpha(e^{\beta L_s} - 1)$$	(A.9)
$$F = F_s$$ $$L_t = L + L_s.$$	(A.10) (A.11)

Ventricular Model

The spherical ventricle model consists of an arrangement of N_c half-sarcomeres on the circumference of a sphere. The number N_c of half-sarcomeres is given by:

$$V_{mw} = K_v L_t^3$$ $$K_v = \frac{N_c^3}{6\pi^2}.$$	(A.12)

The pressure inside the spherical ventricle can be obtained by the following relationship:

$P_{lv} = 5\frac{F}{L_r}\frac{V_w}{K_v L_t^2}.$	(A.13)

To derive the total length of the muscle unit from the ventricle volume V_{lv}, the following equation is used:

$L_t = \sqrt[3]{\frac{V_w f + V_{lv}}{K_v}}.$	(A.14)

Cardiovascular system model

Pressure in the right ventricle is given by:

$P_{rv} = e(t)E_{rv}V_{rv}$	(A.15)

with

$e(t) = \sum_{i=1}^{3} A_i e^{-B_i(t-C_i)^2}.$	(A.16)

The other elastic chambers are passive, hence

$P_{ao} = E_{ao}V_{ao}$	(A.17)
$P_{vc} = E_{vc}V_{vc}$	(A.18)
$P_{pa} = E_{pa}V_{pa}$	(A.19)
$P_{pu} = E_{pu}V_{pu}.$	(A.20)

Flows between chambers are computed by Poiseuilles' law:

$Q_{mt} = \mathrm{r}\left(\frac{P_{pu} - P_{lv}}{R_{mt}}\right)$	(A.21)
$Q_{av} = \mathrm{r}\left(\frac{P_{lv} - P_{ao}}{R_{av}}\right)$	(A.22)
$Q_{sys} = \frac{P_{ao} - P_{vc}}{R_{sys}}$	(A.23)
$Q_{tc} = \mathrm{r}\left(\frac{P_{vc} - P_{rv}}{R_{tc}}\right)$	(A.24)

$$Q_{pv} = \mathrm{r}\left(\frac{P_{rv} - P_{pa}}{R_{ap}}\right) \quad \text{(A.25)}$$

$$Q_{pul} = \frac{P_{pa} - P_{pu}}{R_{pul}} \quad \text{(A.26)}$$

where r denotes the ramp function, defined as

$$\mathrm{r}(x) = \begin{cases} 0, & x < 0 \\ x, & x \geq 0. \end{cases} \quad \text{(A.27)}$$

The volume change in the six chambers can be derived from the continuity equation:

$$\frac{\mathrm{d}V_{lv}}{\mathrm{d}t} = Q_{mt} - Q_{av} \quad \text{(A.28)}$$

$$\frac{\mathrm{d}V_{ao}}{\mathrm{d}t} = Q_{av} - Q_{sys} \quad \text{(A.29)}$$

$$\frac{\mathrm{d}V_{vc}}{\mathrm{d}t} = Q_{sys} - Q_{tc} \quad \text{(A.30)}$$

$$\frac{\mathrm{d}V_{rv}}{\mathrm{d}t} = Q_{tc} - Q_{pv} \quad \text{(A.31)}$$

$$\frac{\mathrm{d}V_{pa}}{\mathrm{d}t} = Q_{pv} - Q_{pul} \quad \text{(A.32)}$$

$$\frac{\mathrm{d}V_{pa}}{\mathrm{d}t} = Q_{pul} - Q_{mt}. \quad \text{(A.33)}$$
